# Supplementary material for: Modeling glioblastoma heterogeneity as a dynamic network of cell states
Source: Mol Syst Biol. 2021 Sep 16;17(9):e10105. doi: 10.15252/msb.202010105 (PMC8444284; doi:10.15252/msb.202010105)
Supplement: Supplementary file 6 — Source Data for Figure 5 [file MSB-17-e10105-s004.zip › Figure5A_sourcedata/GSEA_3017/nosigdb_stateF.GseaPreranked.1622795015644/RICHARDS_ZHONG_NATURE_2018_NPCS_UPREG.html]

Details for gene set RICHARDS\_ZHONG\_NATURE\_2018\_NPCS\_UPREG[GSEA]

|  || Dataset | state33017 |
| Phenotype | NoPhenotypeAvailable |
| Upregulated in class | na\_neg |
| GeneSet | RICHARDS\_ZHONG\_NATURE\_2018\_NPCS\_UPREG |
| Enrichment Score (ES) | -0.82628053 |
| Normalized Enrichment Score (NES) | -5.2893834 |
| Nominal p-value | 0.0 |
| FDR q-value | 0.0 |
| FWER p-Value | 0.0 |
Table: GSEA Results Summary

  

Fig 1: Enrichment plot: RICHARDS\_ZHONG\_NATURE\_2018\_NPCS\_UPREG      
 Profile of the Running ES Score & Positions of GeneSet Members on the Rank Ordered List

  

| PROBE | GENE SYMBOL | GENE\_TITLE | RANK IN GENE LIST | RANK METRIC SCORE | RUNNING ES | CORE ENRICHMENT || 1 | RPS27L |  |  | 33 | 1.025 | -0.0039 | No |
| 2 | HES1 |  |  | 320 | 0.501 | -0.1312 | No |
| 3 | ID4 |  |  | 659 | 0.383 | -0.2840 | No |
| 4 | RCN1 |  |  | 849 | 0.337 | -0.3681 | No |
| 5 | ZFHX4 |  |  | 878 | 0.330 | -0.3774 | No |
| 6 | OTX1 |  |  | 1102 | 0.293 | -0.4777 | No |
| 7 | EFNB1 |  |  | 1152 | 0.285 | -0.4973 | No |
| 8 | CREB5 |  |  | 1561 | -0.275 | -0.6839 | No |
| 9 | APOLD1 |  |  | 1648 | -0.299 | -0.7205 | No |
| 10 | OIP5 |  |  | 1686 | -0.314 | -0.7342 | No |
| 11 | MCM3 |  |  | 1738 | -0.335 | -0.7541 | No |
| 12 | PAICS |  |  | 1757 | -0.342 | -0.7587 | No |
| 13 | WDR34 |  |  | 1806 | -0.365 | -0.7769 | No |
| 14 | LDHA |  |  | 1833 | -0.380 | -0.7847 | No |
| 15 | CDCA4 |  |  | 1852 | -0.391 | -0.7887 | No |
| 16 | UHRF1 |  |  | 1921 | -0.438 | -0.8155 | No |
| 17 | GINS2 |  |  | 1932 | -0.445 | -0.8151 | No |
| 18 | SNRPB |  |  | 1937 | -0.447 | -0.8120 | No |
| 19 | CLIC1 |  |  | 1949 | -0.454 | -0.8121 | No |
| 20 | C19orf48 |  |  | 1979 | -0.474 | -0.8202 | No |
| 21 | MCM4 |  |  | 1993 | -0.487 | -0.8208 | Yes |
| 22 | SPAG5 |  |  | 1997 | -0.490 | -0.8168 | Yes |
| 23 | FEN1 |  |  | 2018 | -0.511 | -0.8204 | Yes |
| 24 | RRM1 |  |  | 2023 | -0.516 | -0.8165 | Yes |
| 25 | DEPDC1B |  |  | 2037 | -0.527 | -0.8166 | Yes |
| 26 | HELLS |  |  | 2038 | -0.528 | -0.8107 | Yes |
| 27 | CDCA3 |  |  | 2041 | -0.529 | -0.8058 | Yes |
| 28 | KIF22 |  |  | 2053 | -0.541 | -0.8048 | Yes |
| 29 | ZWINT |  |  | 2069 | -0.577 | -0.8054 | Yes |
| 30 | BUB3 |  |  | 2078 | -0.596 | -0.8024 | Yes |
| 31 | AURKB |  |  | 2079 | -0.598 | -0.7957 | Yes |
| 32 | CENPK |  |  | 2082 | -0.607 | -0.7899 | Yes |
| 33 | KNSTRN |  |  | 2085 | -0.615 | -0.7839 | Yes |
| 34 | ITGB3BP |  |  | 2102 | -0.652 | -0.7841 | Yes |
| 35 | CKAP2L |  |  | 2109 | -0.663 | -0.7795 | Yes |
| 36 | RPA3 |  |  | 2112 | -0.678 | -0.7729 | Yes |
| 37 | CKS2 |  |  | 2115 | -0.694 | -0.7660 | Yes |
| 38 | CDC20 |  |  | 2117 | -0.695 | -0.7587 | Yes |
| 39 | CENPM |  |  | 2125 | -0.709 | -0.7541 | Yes |
| 40 | CKAP2 |  |  | 2131 | -0.729 | -0.7483 | Yes |
| 41 | KIF2C |  |  | 2136 | -0.756 | -0.7417 | Yes |
| 42 | NDC80 |  |  | 2142 | -0.775 | -0.7354 | Yes |
| 43 | H2AFV |  |  | 2143 | -0.779 | -0.7267 | Yes |
| 44 | FBXO5 |  |  | 2144 | -0.779 | -0.7180 | Yes |
| 45 | KIF23 |  |  | 2145 | -0.782 | -0.7092 | Yes |
| 46 | GMNN |  |  | 2146 | -0.785 | -0.7005 | Yes |
| 47 | MELK |  |  | 2148 | -0.786 | -0.6922 | Yes |
| 48 | DUT |  |  | 2155 | -0.824 | -0.6858 | Yes |
| 49 | MCM7 |  |  | 2160 | -0.839 | -0.6782 | Yes |
| 50 | TUBB4B |  |  | 2162 | -0.843 | -0.6693 | Yes |
| 51 | HMGB3 |  |  | 2164 | -0.852 | -0.6603 | Yes |
| 52 | DTYMK |  |  | 2168 | -0.858 | -0.6521 | Yes |
| 53 | BUB1 |  |  | 2170 | -0.860 | -0.6429 | Yes |
| 54 | CDCA5 |  |  | 2172 | -0.866 | -0.6337 | Yes |
| 55 | NUF2 |  |  | 2176 | -0.877 | -0.6253 | Yes |
| 56 | KPNA2 |  |  | 2178 | -0.894 | -0.6158 | Yes |
| 57 | CCNA2 |  |  | 2181 | -0.902 | -0.6067 | Yes |
| 58 | MIS18BP1 |  |  | 2183 | -0.903 | -0.5971 | Yes |
| 59 | KIF15 |  |  | 2185 | -0.915 | -0.5873 | Yes |
| 60 | PRR11 |  |  | 2187 | -0.930 | -0.5774 | Yes |
| 61 | ORC6 |  |  | 2188 | -0.937 | -0.5670 | Yes |
| 62 | C21orf58 |  |  | 2189 | -0.937 | -0.5565 | Yes |
| 63 | CENPW |  |  | 2192 | -0.943 | -0.5469 | Yes |
| 64 | TUBA1B |  |  | 2193 | -0.947 | -0.5363 | Yes |
| 65 | HJURP |  |  | 2195 | -0.962 | -0.5261 | Yes |
| 66 | UBE2T |  |  | 2196 | -0.965 | -0.5153 | Yes |
| 67 | CDKN3 |  |  | 2199 | -0.979 | -0.5053 | Yes |
| 68 | CCNB2 |  |  | 2200 | -0.983 | -0.4943 | Yes |
| 69 | CKS1B |  |  | 2201 | -0.987 | -0.4833 | Yes |
| 70 | SMC2 |  |  | 2205 | -1.006 | -0.4735 | Yes |
| 71 | KIF11 |  |  | 2206 | -1.008 | -0.4622 | Yes |
| 72 | SPC25 |  |  | 2208 | -1.019 | -0.4513 | Yes |
| 73 | DHFR |  |  | 2212 | -1.040 | -0.4411 | Yes |
| 74 | CLSPN |  |  | 2216 | -1.060 | -0.4306 | Yes |
| 75 | CENPN |  |  | 2217 | -1.079 | -0.4186 | Yes |
| 76 | UBE2C |  |  | 2218 | -1.081 | -0.4065 | Yes |
| 77 | CENPU |  |  | 2219 | -1.088 | -0.3944 | Yes |
| 78 | H2AFZ |  |  | 2220 | -1.090 | -0.3822 | Yes |
| 79 | H2AFX |  |  | 2223 | -1.146 | -0.3703 | Yes |
| 80 | CCNB1 |  |  | 2224 | -1.148 | -0.3575 | Yes |
| 81 | CDK1 |  |  | 2225 | -1.153 | -0.3446 | Yes |
| 82 | PTTG1 |  |  | 2226 | -1.198 | -0.3313 | Yes |
| 83 | ANP32E |  |  | 2227 | -1.200 | -0.3179 | Yes |
| 84 | MAD2L1 |  |  | 2228 | -1.204 | -0.3044 | Yes |
| 85 | GTSE1 |  |  | 2230 | -1.220 | -0.2913 | Yes |
| 86 | HMGN2 |  |  | 2233 | -1.267 | -0.2781 | Yes |
| 87 | RRM2 |  |  | 2234 | -1.268 | -0.2639 | Yes |
| 88 | DLGAP5 |  |  | 2235 | -1.273 | -0.2497 | Yes |
| 89 | SMC4 |  |  | 2236 | -1.281 | -0.2354 | Yes |
| 90 | PBK |  |  | 2237 | -1.325 | -0.2206 | Yes |
| 91 | ATAD2 |  |  | 2238 | -1.381 | -0.2052 | Yes |
| 92 | TMPO |  |  | 2239 | -1.382 | -0.1898 | Yes |
| 93 | PRC1 |  |  | 2240 | -1.397 | -0.1742 | Yes |
| 94 | HMGB2 |  |  | 2241 | -1.469 | -0.1578 | Yes |
| 95 | BIRC5 |  |  | 2242 | -1.469 | -0.1413 | Yes |
| 96 | ASPM |  |  | 2244 | -1.490 | -0.1252 | Yes |
| 97 | TOP2A |  |  | 2246 | -1.562 | -0.1082 | Yes |
| 98 | NUSAP1 |  |  | 2247 | -1.600 | -0.0903 | Yes |
| 99 | TPX2 |  |  | 2248 | -1.609 | -0.0724 | Yes |
| 100 | CENPF |  |  | 2249 | -1.691 | -0.0535 | Yes |
| 101 | MKI67 |  |  | 2252 | -1.892 | -0.0333 | Yes |
| 102 | HIST1H4C |  |  | 2253 | -2.984 | -0.0000 | Yes |
Table: GSEA details [plain text format]

  

Fig 2: RICHARDS\_ZHONG\_NATURE\_2018\_NPCS\_UPREG: Random ES distribution      
 Gene set null distribution of ES for **RICHARDS\_ZHONG\_NATURE\_2018\_NPCS\_UPREG**
